# Supplementary material for: Post-click labeling enables highly accurate single cell analyses of glucose uptake ex vivo and in vivo
Source: Commun Biol. 2024 Apr 16;7:459. doi: 10.1038/s42003-024-06164-y (PMC11021395; doi:10.1038/s42003-024-06164-y)
Supplement: Supplementary file 2 — Supplementary Information [file 42003_2024_6164_MOESM2_ESM.pdf]

Supplementary information

Post-click labeling enables highly accurate single cell analyses of glucose uptake *ex vivo* and *in vivo*

Masaki Tsuchiya, Nobuhiko Tachibana, and Itaru Hamachi

This file contains Supplementary Figures S1-S6.

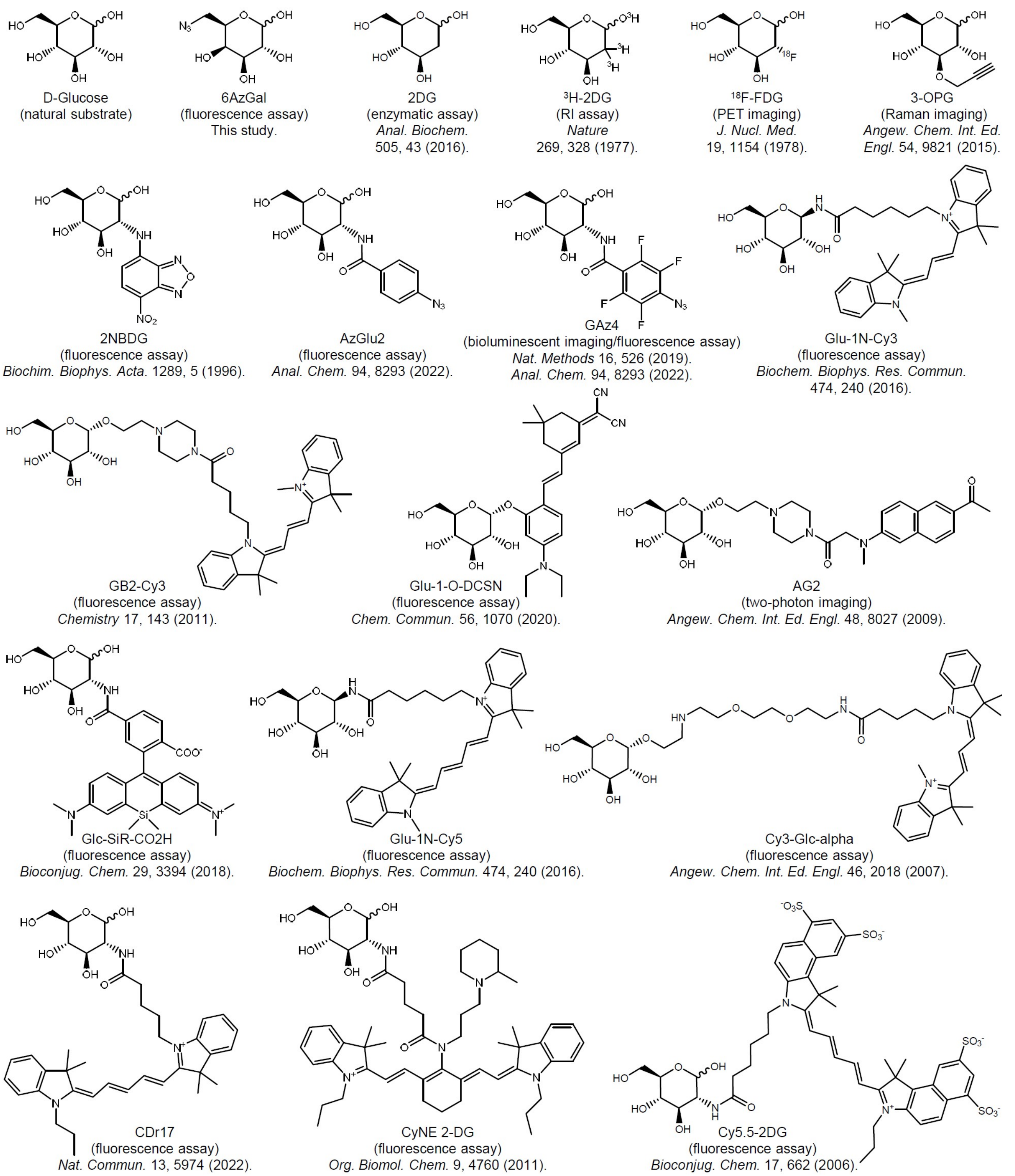

Fig. S1. Structures of glucose analogs. Structures of D-glucose, 6AzGal, and existing glucose uptake probes are shown with their applications and references.

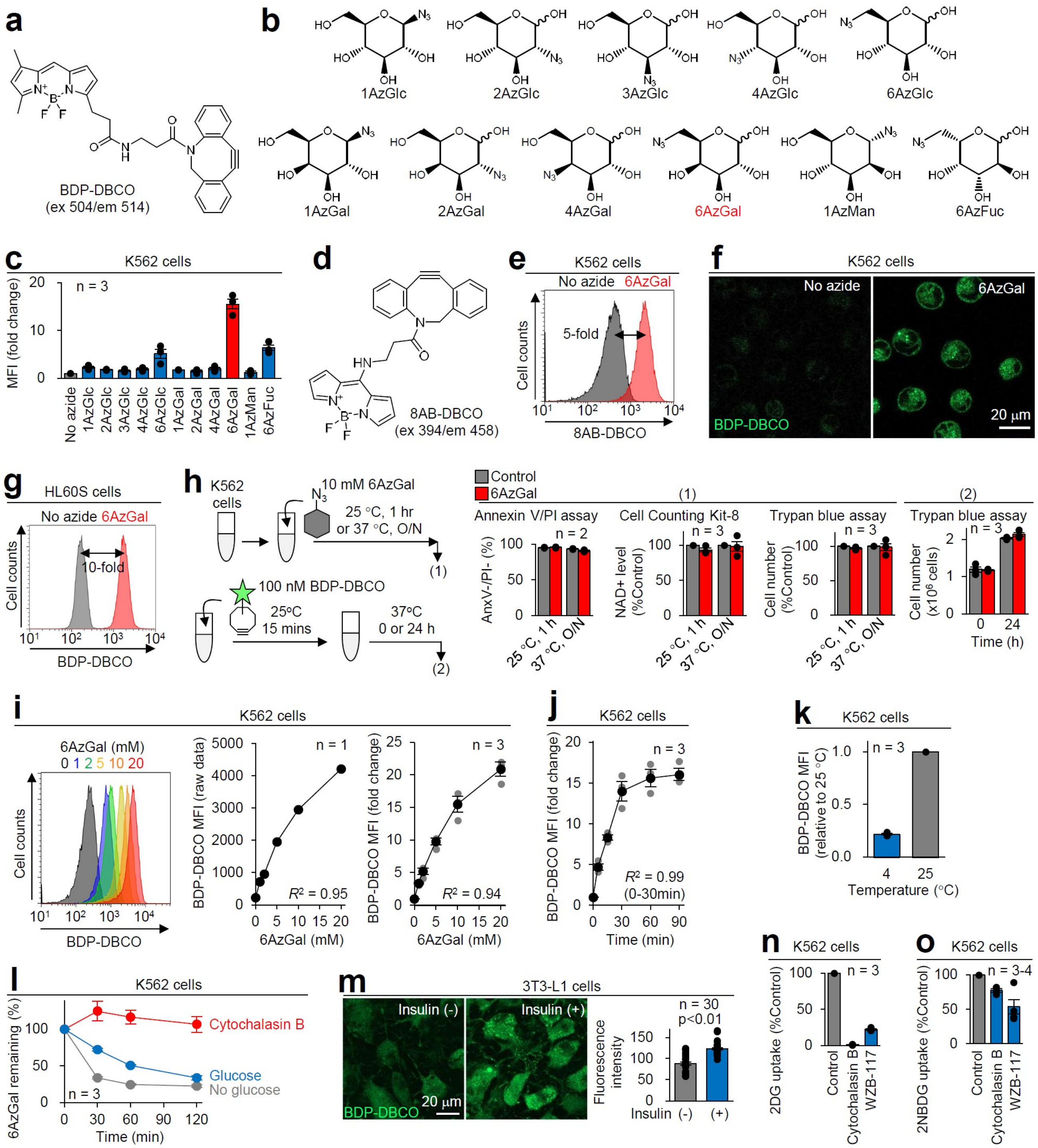

Fig. S2. Supporting data for measurement of 6AzGal uptake *in vitro*. (a) Structure of BDP-DBCO. (b) Structures of azido-sugars tested in this study. (c) Flow cytometric quantification of azido-sugar uptake in K562 cells by MFIs of BDP-DBCO labeling. (d) Structure of 8AB-DBCO. (e) Flow cytometric analysis of 6AzGal uptake in K562 cells by 8AB-DBCO labeling. (f) Confocal imaging of BDP-DBCO-labeled 6AzGal in K562 cells. (g) Flow cytometric analysis of 6AzGal uptake in HL60S cells by BDP-DBCO labeling. (h) Viability assays showing no obvious toxicity of 6AzGal treatment or BDP-DBCO labeling in K562 cells. (i) Concentration-dependent uptake of 6AzGal in K562 cells. Cells treated with various 6AzGal amounts for 1 h at 25 °C were labeled with BDP-DBCO and analyzed by flow cytometry (Left: representative histogram. Center: dot plot

using raw MFIs. Right: dot plot using MFI fold changes). (j) Uptake kinetics of 6AzGal in K562 cells. Cells treated with 10 mM 6AzGal for various periods at 25 °C were labeled with BDP-DBCO and analyzed by flow cytometry. (k) Temperature-dependent uptake of 6AzGal in K562 cells. Cells treated with 10 mM 6AzGal at 4 or 25 °C were labeled with BDP-DBCO and analyzed by flow cytometry. (l) Efflux kinetics of 6AzGal in the presence of glucose and cytochalasin B. 6AzGal-loaded K562 cells were incubated without glucose, with glucose, and with cytochalasin B for the indicated periods, followed by flow cytometric quantification of BDP-DBCO labeling. (m) Confocal imaging and quantification of BDP-DBCO-labeled 6AzGal in differentiated 3T3-L1 adipocytes treated with insulin. (n) Potent inhibition of 2DG uptake by GLUT inhibitors. K562 cells were treated with 2DG in the presence of cytochalasin B and WZB-117, and subjected to the Glucose Uptake-Glo Assay (Promega). (o) Weak inhibition of 2NBDG uptake by GLUT inhibitors. K562 cells were treated with 2NBDG in the presence of cytochalasin B and WZB-117, followed by flow cytometric analysis. Bar graphs represent means ± SEM. *P*-values were determined by the t-test.

	Channel	FL1	FL2	FL3	FL4	FL5	FL6	FL7	FL8	FL9	FL10	FL11	FL12
	Excitation laser (nm)	488&561					405&638						
	Band path filter (nm)	525/50	585/30	617/30	695/50	785/60	450/50	525/50	585/30	617/30	665/30	720/60	785/60
Fluorophore	BDP	100	8	3	0	0	0	1	0	0	0	0	0
	AF488	100	17	6	1	0	0	1	0	0	0	0	0
	Fluorescein	100	20	11	3	1	0	3	1	1	0	0	0
	PE	0	100	45	6	1	0	0	2	1	0	0	0
	mCherry	0	33	100	53	16	0	0	0	4	1	1	0
	PE/Dazzle594	0	22	100	31	4	0	0	0	2	0	0	0
	PE/Cy5	0	2	1	100	14	0	0	0	0	14	8	1
	PerCP/Cy5.5	3	0	5	100	39	1	0	9	6	20	53	11
	PE/Cy7	0	1	0	0	100	0	0	0	0	0	0	2
	BV421	0	0	0	0	0	100	7	0	0	0	0	0
	CPM	0	0	0	0	0	100	43	5	2	0	0	0
	FVD450	0	0	0	0	0	100	11	1	0	0	0	0
	FVD506	1	0	0	0	0	23	100	23	10	1	1	0
	BV510	3	2	1	1	0	18	100	34	19	4	3	1
	AF647	0	0	0	9	2	0	0	0	0	100	58	10
	AF700	0	0	2	7	7	1	0	3	2	2	100	14
	BV711	0	0	1	9	8	7	1	4	3	3	100	15
	FVD780	0	0	0	0	38	0	0	0	0	1	4	100

Relative fluorescence intensity (%)

0 50 100

Fig. S3. Fluorescent spectral dataset for multicolor flow cytometric analysis. The table shows relative fluorescence intensities of fluorophores detected by the Sony MA900 cell sorter equipped with 12 channels. For minimal spectral overlapping with BDP-DBCO, fluorophores with a relative fluorescence intensity lower than 1% in the FL1 channel were used in multicolor assays.

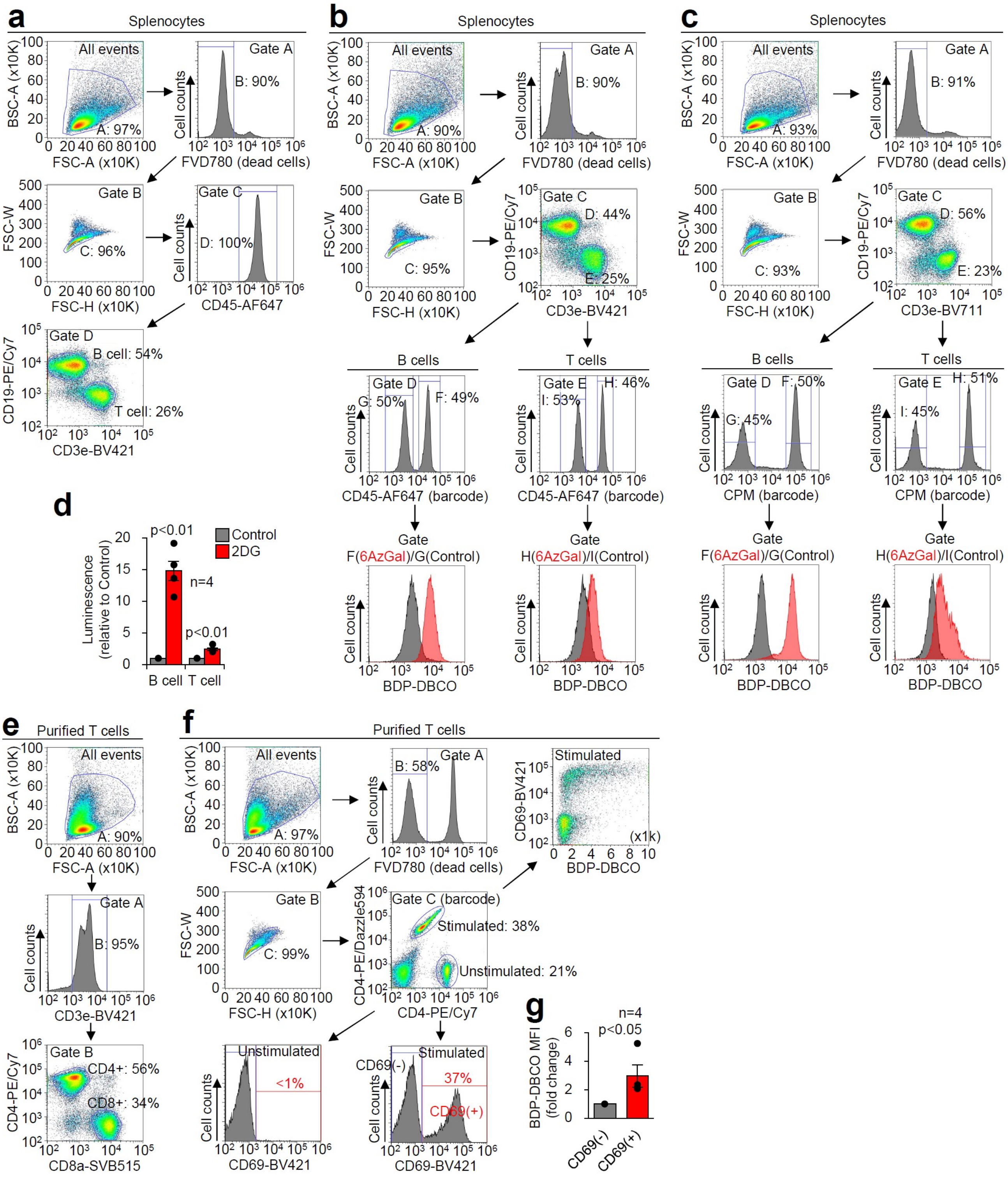

Fig. S4. Supporting data for measurement of 6AzGal uptake *ex vivo*. (a) Gating strategy to separate splenic B and T cells. Mouse splenocytes were labeled with antibodies against CD45, CD19, and CD3e in combination with FVD780 (dead cell marker). Using forward scatter (FSC) and back scatter (BSC) in all events, live cells were distinguished from cell debris and dead cells. In Gate A, live cells were confirmed by negative staining with FVD780. In Gate B, singlet cells were separated from doublet cells by physical parameters (FSC-H vs. FSC-W). In Gate C, all cells were $CD45^+$ lymphocytes. In Gate D, $CD19^+$ B cells and $CD3e^+$ T cells were separated. (b) Gating

strategy to measure 6AzGal uptake in splenic B and T cells with antibody barcoding. Control and 6AzGal-treated splenocytes were labeled with antibodies, BDP-DBCO, and FVD780. For barcoding, an anti-CD45 antibody was added only to the 6AzGal-treated sample. Control and 6AzGal-treated samples were pooled and analyzed by flow cytometry. Singlet cells (Gate C) were gated for B cells (Gate D) and T cells (Gate E), followed by separation of control and 6AzGal-treated cells by CD45 levels (Gate F–I). (c) Gating strategy to measure 6AzGal uptake in splenic B and T cells with CPM barcoding. The experiment was conducted as described in (b), but CPM was used instead of the anti-CD45 antibody for barcoding. (d) Bulk measurements of 2DG uptake in B and T cells. B and T cells were purified from mouse splenocytes by EasySep isolation kits (STEMCELL Technologies). The cells were treated with 2DG and subjected to the Glucose Uptake-Glo Assay (Promega). (e) Gating strategy to separate $CD4^+$ and $CD8^+$ T cells. T cells isolated from mouse splenocytes were labeled with antibodies against CD3e, CD4, and CD8a, and analyzed by flow cytometry, confirming the purity of the T cell population. (f) Gating strategy to measure 6AzGal uptake in activated $CD4^+$ T cells. Purified T cells were incubated with Dynabeads Mouse T-Activator CD3/CD28 (Thermo Fisher), loaded with 6AzGal, and labeled with antibodies, FVD780, and BDP-DBCO, followed by flow cytometry. For barcoding, stimulated and unstimulated T cells were stained with PE/Dazzle594 and PE/Cy7, respectively. In the dot plot of stimulated $CD4^+$ cells (top right), subpopulations were divided by the order of the BDP-DBCO level to evaluate the correlation between CD69 expression and BDP-DBCO labeling (Fig. 4c, right). (g) Quantification of 6AzGal uptake in activated $CD4^+$ T cells (Fig. 4c, middle). Bar graphs represent means ± SEM. *P*-values were determined by the t-test.

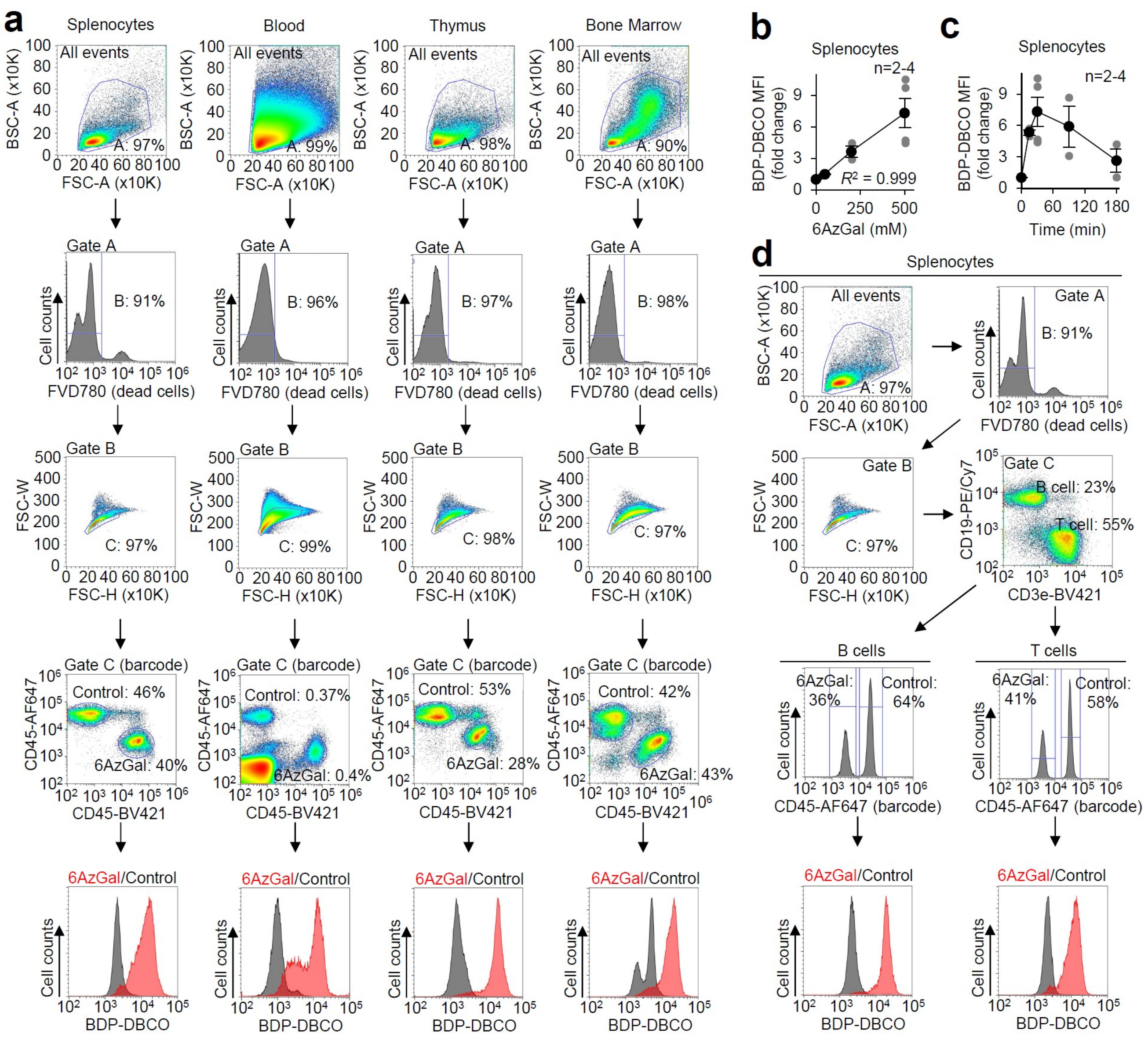

Fig. S5. Supporting data for measurement of 6AzGal uptake *in vivo*. (a) Gating strategy to measure 6AzGal uptake in the spleen, blood, thymus, and bone marrow. 6AzGal (20 mg/kg) was intraperitoneally administered to fasted mice. Thirty minutes later, tissues were harvested. Cells were dissociated and labeled with antibodies, FVD780, and BDP-DBCO. For barcoding, different fluorescently labeled anti-CD45 antibodies were applied to control cells (AF647) and 6AzGal-treated cells (BV421). Singlet live cells were separated by CD45 gating to compare BDP-DBCO labeling between control and 6AzGal-treated samples. (b) Concentration-dependent uptake of 6AzGal in the spleen. Thirty minutes after administration of various 6AzGal amounts, leukocytes isolated from the spleen were labeled with BDP-DBCO and analyzed by flow cytometry. (c) Uptake kinetics of 6AzGal in the spleen. At the indicated times after 6AzGal injection, splenic leukocytes were isolated and subjected to quantification of BDP-labeled 6AzGal. (d) Gating strategy to measure 6AzGal uptake in splenic B and T cells. After 6AzGal injection, isolated splenocytes were labeled with antibodies, BDP-DBCO, and FVD780. An AF647-conjugated anti-CD45 antibody was used for barcoding. Live singlet cells were gated for B and T cells, separated by CD45 levels, and analyzed for BDP-DBCO labeling.

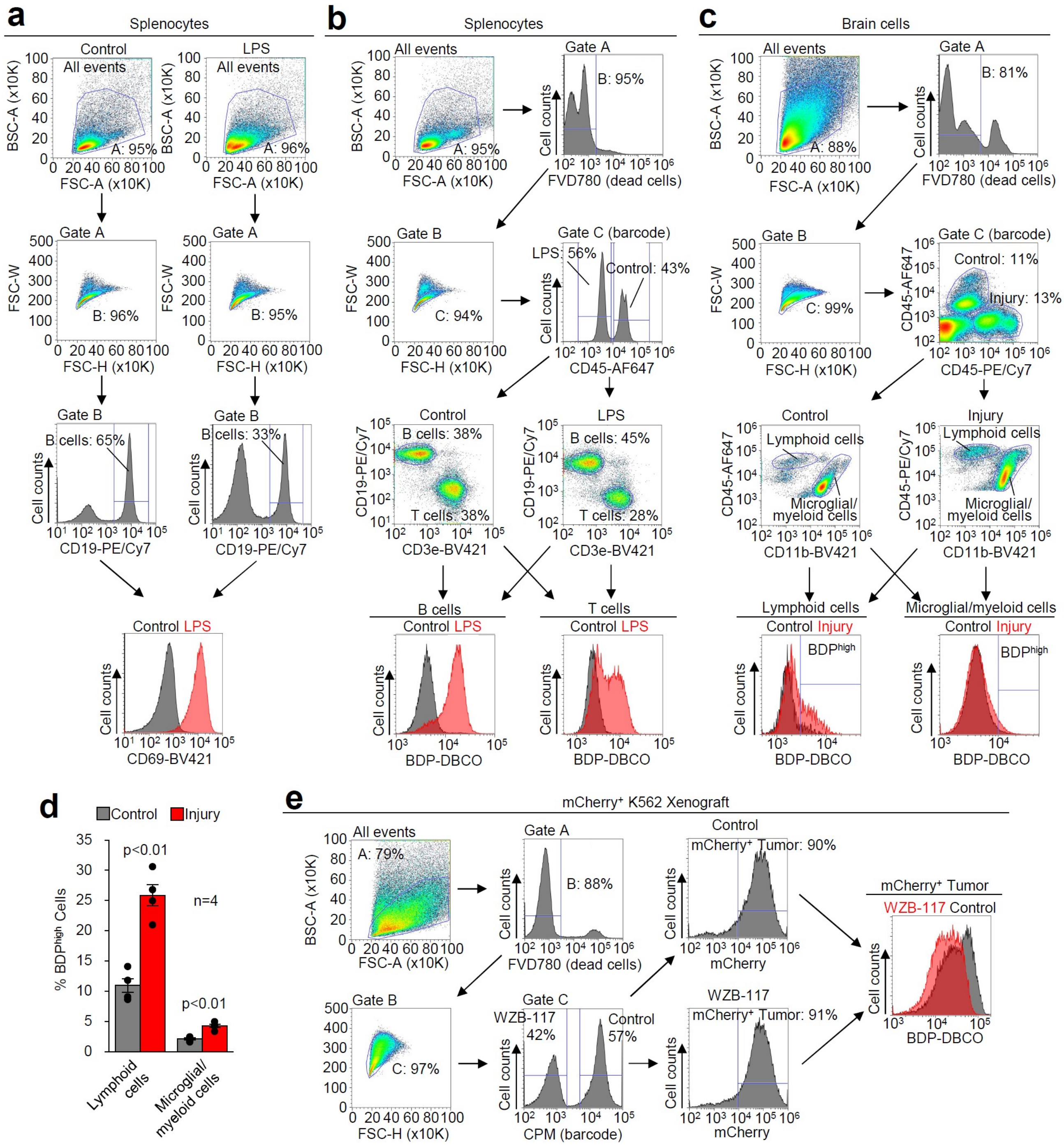

Fig. S6. Supporting data for measurement of 6AzGal uptake in disease-associated mouse models. (a) Gating strategy to confirm LPS-stimulated activation of splenic B cells. LPS (50 mg/kg) was intraperitoneally administered to mice. Four hours later, splenocytes were harvested. Cells were labeled with antibodies against CD19 and CD69, and analyzed by flow cytometry. Singlet cells (Gate B) were gated for CD19^{+} B cells to compare CD69 expression levels between control and LPS-treated samples. (b) Gating strategy to measure 6AzGal uptake in LPS-activated splenic B and T cells. After injection of 6AzGal into LPS-treated mice, isolated splenocytes were labeled with antibodies, FVD780, and BDP-DBCO, and analyzed by flow cytometry. Control and LPS-treated cells were distinguished by CD45 barcoding, followed by separation of B and T cells to quantify BDP-DBCO-labeled 6AzGal. (c, d) Gating strategy to measure 6AzGal uptake in brain immune cells upon ischemic stroke injury. After

mice subjected to surgically induced cerebral infraction were treated by retro-orbital injection of 6AzGal, brain cells were collected, labeled with antibodies, FVD780, and BDP-DBCO, and analyzed by flow cytometry. Live singlet cells were distinguished by CD45 barcoding [control (AF647) vs. injured cells (PE/Cy7)], followed by separation of lymphoid cells ($CD45^{high}/CD11b^{low}$) and microglial ($CD45^{low}/CD11b^{intermediate}$)/myeloid ($CD45^{high}/CD11b^{high}$) cells to quantify BDP-DBCO-labeled 6AzGal (c). In the bottom panels, the percentages of subpopulations with higher levels of BDP-DBCO fluorescence were quantified for comparison between control and injury samples (d). (e) Gating strategy to measure 6AzGal uptake in K562 xenografts treated with WZB-117. Nude mice with mCherry-expressing K562 xenografts on the dorsal subcutis were treated with WZB-117 for 1 h, followed by 6AzGal injection. Cells collected from tumors were labeled with CPM (barcode for control samples), FVD780, and BDP-DBCO, and analyzed by flow cytometry. Live singlet cells (Gate C) were gated for CPM^+ (Control) and CPM^- (WZB-117), followed by selection of $mCherry^+$ K562 cells to quantify BDP-DBCO-labeled 6AzGal. Bar graphs represent means ± SEM. *P*-values were determined by the t-test.
