# Supplementary material for: Post-click labeling enables highly accurate single cell analyses of glucose uptake ex vivo and in vivo
Source: Commun Biol. 2024 Apr 16;7:459. doi: 10.1038/s42003-024-06164-y (PMC11021395; doi:10.1038/s42003-024-06164-y)
Supplement: Supplementary file 5 — Reporting Summary [file 42003_2024_6164_MOESM5_ESM.pdf]

nature portfolio

Corresponding author(s): Itaru Hamachi

Last updated by author(s): Aug 18, 2023

Reporting Summary

Nature Portfolio wishes to improve the reproducibility of the work that we publish. This form provides structure for consistency and transparency in reporting. For further information on Nature Portfolio policies, see our Editorial Policies and the Editorial Policy Checklist.

Statistics

For all statistical analyses, confirm that the following items are present in the figure legend, table legend, main text, or Methods section.

n/a	Confirmed	
☐	☒	The exact sample size (*n*) for each experimental group/condition, given as a discrete number and unit of measurement
☐	☒	A statement on whether measurements were taken from distinct samples or whether the same sample was measured repeatedly
☐	☒	The statistical test(s) used AND whether they are one- or two-sided<br>*Only common tests should be described solely by name; describe more complex techniques in the Methods section.*
☒	☐	A description of all covariates tested
☒	☐	A description of any assumptions or corrections, such as tests of normality and adjustment for multiple comparisons
☐	☒	A full description of the statistical parameters including central tendency (e.g. means) or other basic estimates (e.g. regression coefficient) AND variation (e.g. standard deviation) or associated estimates of uncertainty (e.g. confidence intervals)
☐	☒	For null hypothesis testing, the test statistic (e.g. *F*, *t*, *r*) with confidence intervals, effect sizes, degrees of freedom and *P* value noted<br>*Give P values as exact values whenever suitable.*
☒	☐	For Bayesian analysis, information on the choice of priors and Markov chain Monte Carlo settings
☒	☐	For hierarchical and complex designs, identification of the appropriate level for tests and full reporting of outcomes
☒	☐	Estimates of effect sizes (e.g. Cohen's *d*, Pearson's *r*), indicating how they were calculated

Our web collection on statistics for biologists contains articles on many of the points above.

Software and code

Policy information about availability of computer code

Data collection	No software was used
Data analysis	No software was used

For manuscripts utilizing custom algorithms or software that are central to the research but not yet described in published literature, software must be made available to editors and reviewers. We strongly encourage code deposition in a community repository (e.g. GitHub). See the Nature Portfolio guidelines for submitting code & software for further information.

Data

Policy information about availability of data

All manuscripts must include a data availability statement. This statement should provide the following information, where applicable:

- Accession codes, unique identifiers, or web links for publicly available datasets
- A description of any restrictions on data availability
- For clinical datasets or third party data, please ensure that the statement adheres to our policy

Any additional information required to reanalyze the data reported in this paper is available upon request.

Research involving human participants, their data, or biological material

Policy information about studies with human participants or human data. See also policy information about sex, gender (identity/presentation), and sexual orientation and race, ethnicity and racism.

Reporting on sex and gender	No human participants or human data were used in this study.
Reporting on race, ethnicity, or other socially relevant groupings	No human participants or human data were used in this study.
Population characteristics	No human participants or human data were used in this study.
Recruitment	No human participants or human data were used in this study.
Ethics oversight	No human participants or human data were used in this study.

Note that full information on the approval of the study protocol must also be provided in the manuscript.

Field-specific reporting

Please select the one below that is the best fit for your research. If you are not sure, read the appropriate sections before making your selection.

☒ Life sciences ☐ Behavioural & social sciences ☐ Ecological, evolutionary & environmental sciences

For a reference copy of the document with all sections, see nature.com/documents/nr-reporting-summary-flat.pdf

Life sciences study design

All studies must disclose on these points even when the disclosure is negative.

Sample size	No sample-size calculation was performed in this study. For any flow cytometric analyses, at least 30,000 sample sizes were analyzed for each replicate.
Data exclusions	No data were excluded in this study.
Replication	We collected at least 3 replicates for each experiment to verify the reproducibility of the experimental findings.
Randomization	For all experiments, cells and mice were randomly allocated.
Blinding	No blinding was used for group allocation, as samples were randomly allocated.

Reporting for specific materials, systems and methods

We require information from authors about some types of materials, experimental systems and methods used in many studies. Here, indicate whether each material, system or method listed is relevant to your study. If you are not sure if a list item applies to your research, read the appropriate section before selecting a response.

Materials & experimental systems

n/a	Involved in the study	
☐	☒	Antibodies
☐	☒	Eukaryotic cell lines
☒	☐	Palaeontology and archaeology
☐	☒	Animals and other organisms
☒	☐	Clinical data
☒	☐	Dual use research of concern
☒	☐	Plants

Methods

n/a	Involved in the study	
☒	☐	ChIP-seq
☐	☒	Flow cytometry
☒	☐	MRI-based neuroimaging

Antibodies

Antibodies used	CD19-PE/Cy7 (Supplier: BioLegend; Identifier: 115519; Clone: 6D5); CD3e-BV421 (Supplier: BioLegend; Identifier: 100335; Clone: 145-2C11); CD3e-BV711(Supplier: BioLegend; Identifier: 100349; Clone: 145-2C11); CD4-PE/Cy7(Supplier: BioLegend; Identifier: 100527; Clone: RM4-5); CD4-PE/Dazzle594(Supplier: BioLegend; Identifier: 100565; Clone: RM4-5); CD8a-SVB515(Supplier: BIO-RAD;

Identifier: MCA609SBV515; Clone: KT15); CD69-BV421(Supplier: BioLegend; Identifier: 104527; Clone: H1.2F3); CD45-AF647(Supplier: BioLegend; Identifier: 103123; Clone: 30-F11); CD45-BV421(Supplier: BioLegend; Identifier: 103133; Clone: 30-F11); CD11b-BV421(Supplier: BioLegend; Identifier: 101235; Clone: M1/70)

Validation

All antibodies used in this study were verified to react against mouse immunogen and fully compatible for flow cytometry, according to the manufacturer's website.

Eukaryotic cell lines

Policy information about cell lines and Sex and Gender in Research

Cell line source(s)	K562 (Supplier: Riken BRC; Identifier: RCB0027); HL60S (Supplier: JCRB; Identifier: JCRB0163); HCC1806 (Supplier: ATCC; Identifier: CRL-2335); 3T3-L1 MBX (Supplier: ATCC; Identifier: CRL-3242).
Authentication	None of the cell lines used were authenticated.
Mycoplasma contamination	All cell lines were tested negative for mycoplasma contamination.
Commonly misidentified lines (See ICLAC register)	N/A

Animals and other research organisms

Policy information about studies involving animals; ARRIVE guidelines recommended for reporting animal research, and Sex and Gender in Research

Laboratory animals	Mice (Species: Mus Musculus; Age:6-8 weeks; Strain: C57BL/6J, CBALB/c Slc-nu, C.B-17/Icr-scid/scidJcl)
Wild animals	The study did not involve any wild animals.
Reporting on sex	Although only female mice were used in this study, the sex was not considered in study design and methods used. The main and sole purpose of this paper is to introduce a new technique. Evaluating differences between male and female is out of the scope of this study.
Field-collected samples	The study did not involve samples collected from the field.
Ethics oversight	All animal husbandry and experimental procedures were approved by the Animal Care Use and Review Committee of Kyoto University.

Note that full information on the approval of the study protocol must also be provided in the manuscript.

Flow Cytometry

Plots

Confirm that:

- [x] The axis labels state the marker and fluorochrome used (e.g. CD4-FITC).
- [x] The axis scales are clearly visible. Include numbers along axes only for bottom left plot of group (a 'group' is an analysis of identical markers).
- [x] All plots are contour plots with outliers or pseudocolor plots.
- [x] A numerical value for number of cells or percentage (with statistics) is provided.

Methodology

Sample preparation

For all established cell lines (K562, HL60S, HCC1806), fluorescent-labeled cells were resuspended in 10%FBS/IMDM with cell density adjusted to 5 - 10 x 10^5 cells/mL and minimum volume of 200 uL. Cells were filtered and transfered into a 5 ml tube with cell strainer to remove any clustering cells.
For ex vivo azido-sugar uptake analysis of splenocytes, spleens were collected from euthanized mice, dilacerated and filtered through a 70um cell strainer, treated with VersaLyse lysis buffer to remove red blood cells. Collected cells were subjected to Azido-sugar incorporation, followed by fluorescence click chemistry and immunolabeling. Fluorescent-labeled spenocytes were washed with 10% FBS/IMDM twice, then resuspended in 10% FBS/IMDM with cell density adjusted to 5 - 10 x 10^5 cells/mL and minimum volume of 200 uL. For T cell activation, T cells were isolated from the single cell suspension of splenocytes with a kit. Purified T cells were cultured with Mouse T-activator CD3/CD28 for 48 hours, then further processes as described above.
For in vivo azido-sugar uptake assay, azido-sugar was administered intraperitoneally or retro-orbitally into fasted mice. Single cell suspensions of spleen and thymus were prepared as described above. Whole blood was collected by cardiac puncture in heparin tubes, treated with VersaLyse buffer. Bone marrow-derived cells was collected from femur and tibia of the mice, and treated with VersaLyse buffer. Microglia were isolated from the rodent forebrain using Percoll gradient. Isolated cells were then labelled with fluorescence clock chemistry and immunolabeling, washed with 10% FBS/IMDM twice, then resuspended in 10% FBS/IMDM with cell density adjusted to 5 - 10 x 10^5 cells/mL and minimum volume of 200 uL.

Instrument | Maker: Sony; Model: Cell Sorter MA900

Software | Cell Sorter Software by Sony

Cell population abundance | No cell sorting was performed in this study.

Gating strategy

Scatter density plot (FSC-A vs BSC-A): For K562 cells, typically live cells lie within the range of 20 – 70 (x10,000) in FSC-A and 10 – 40 (x10,000) in BSC-A . Events with <20 (x10,000) in FSC-A are either debris or dead cells and were gated out. Any events that lie >70 (x10,000) in FSC-A and >40 (x10,000) in BSC-A are likely non-singlets and were avoided.

Scatter density plot (FSC-H vs FSC-W): Gated for an area where majority (90 – 100%) of events reside, and were considered "single cell".

Fluorescence detection: Prior to the analyses, non-fluorescence "negative" cells were prepared (For glucose uptake: no azido-sugar, BDP-labeled cells; For fluorescence immunolabeling to identify cell type: cells labeled with fluorescence-conjugated non-specific IgG), ran on flow cytometer, and for each corresponding fluorescence detector, sensor gain were adjusted so the detected cell population lie below 2x10^3. Instrumental settings for each cell types were shown in the method section. Any cells lie above 2x10^3 were considered "positive".

Cell viability: Prior to the analyses, unlabeled cells were used to adjust the sensor gain for FVD780. Any cells lies below 2x10^3 were considered "negative" live cells, and any cells with fluorescence signal above 2x10^3 were considered "positive" for dead cells. To validate the quality of FVD780 dyes, cells were boiled at 90 degree Celsius for 2 mins, stained with FVD780, and verified that all dead cells were stained.

☒ Tick this box to confirm that a figure exemplifying the gating strategy is provided in the Supplementary Information.
